# A systematic review of clinical efficacy and safety of cell-based therapies in Alzheimer’s disease

**DOI:** 10.1590/1980-5764-DN-2024-0147

**Published:** 2024-09-06

**Authors:** Hamidreza Feizi, Mohammad-Salar Hosseini, Sepideh Seyedi-Sahebari, Hanie Karimi, Reza Mosaddeghi-Heris, Saeed Sadigh-Eteghad, Fatemeh Sadeghi-Ghyassi, Mahnaz Talebi, Amirreza Naseri, Hanieh Salehi-Pourmehr, Leila Roshangar

**Affiliations:** 1 Tabriz University of Medical Sciences, Student Research Committee, Tabriz, Iran. Tabriz University of Medical Sciences Student Research Committee Tabriz Iran; 2 Tabriz University of Medical Sciences, Aging Research Institute, Research Center for Integrative Medicine in Aging, Tabriz, Iran. Tabriz University of Medical Sciences Aging Research Institute Research Center for Integrative Medicine in Aging Tabriz Iran; 3 Tabriz University of Medical Sciences, Research Center for Evidence-Based Medicine, Iranian EBM Centre: JBI Centre of Excellence, Faculty of Medicine, Tabriz, Iran. Tabriz University of Medical Sciences Research Center for Evidence-Based Medicine Iranian EBM Centre: JBI Centre of Excellence, Faculty of Medicine Tabriz Iran; 4 Tehran University of Medical Sciences, School of Medicine, Tehran, Iran. Tehran University of Medical Sciences School of Medicine Tabriz Iran; 5 Tabriz University of Medical Sciences, Neuroscience Research Center, Tabriz, Iran. Tabriz University of Medical Sciences Neuroscience Research Center Tabriz Iran; 6 Tabriz University of Medical Sciences, Tabriz Valiasr Hospital, Clinical Research Development Unit, Tabriz, Iran. Tabriz University of Medical Sciences Tabriz Valiasr Hospital Clinical Research Development Unit Tabriz Iran; 7 Tabriz University of Medical Sciences, Medical Philosophy and History Research Center, Tabriz, Iran. Tabriz University of Medical Sciences Medical Philosophy and History Research Center Tabriz Iran; 8 Tabriz University of Medical Sciences, Stem Cell Research Center, Tabriz, Iran. Tabriz University of Medical Sciences Stem Cell Research Center Tabriz Iran

**Keywords:** Alzheimer Disease, Cell Transplantation, Stem Cell Transplantation, Systematic Review, Doença de Alzheimer, Transplante de Células, Transplante de Células-Tronco, Revisão Sistemática

## Abstract

There is presently no disease-modifying therapy for Alzheimer’s Disease (AD), which is the most prevalent cause of dementia.

**Objective:**

This study aspires to estimate the efficacy and safety of cell-based treatments in AD.

**Methods:**

Observing the Joanna Briggs Institute (JBI) methods and Preferred Reporting Items for Systematic Reviews and Meta-Analyses (PRISMA) statement, a systematic search was accomplished in PubMed, Medical Literature Analysis and Retrieval System Online (Medline, via Ovid), Embase; Cochrane, and Cumulative Index of Nursing and Allied Health Literature — CINAHL (via EBSCO) databases up to June 2023. The relevant clinical studies in which cell-based therapies were utilized to manage AD were included. The risk of bias was evaluated using the JBI checklists, based on the study designs.

**Results:**

Out of 1,014 screened records, a total of five studies with 70 individuals (including 59 patients receiving stem cells and 11 placebo controls) were included. In all these studies, despite the discrepancy in the origin of stem cells, cell density, and transplant site, safety goals were obtained. The intracerebroventricular injection of adipose-derived stromal vascular fraction (ADSVF) and umbilical cord-derived mesenchymal stem cells (UC-MSCs), the intravenous injection of Lomecel-B, and the bilateral hippocampi and right precuneus injection of UC-MSCs are not linked to any significant safety concerns, according to the five included studies. Studies also revealed improvements in biomarkers and clinical outcomes as a secondary outcome. Three studies had no control groups and there are concerns regarding the similarity of the groups in others. Also, there is considerable risk of bias regarding the outcome assessment scales.

**Conclusion:**

Cell-based therapies are well tolerated by AD patients, which emphasizes the need for further, carefully planned randomized studies to reach evidence-based clinical recommendations in this respect.

## RESUMO

Atualmente, não há terapia modificadora da doença para a doença de Alzheimer (DA), que é a causa mais prevalente de demência.

**Objetivo:**

Este estudo teve como objetivo estimar a eficácia e segurança dos tratamentos baseados em células na DA.

**Métodos:**

Observando os métodos do JBI e a declaração PRISMA, uma busca sistemática foi realizada nas bases de dados PubMed, Medical Literature Analysis and Retrieval System Online — Medline (via Ovid), Embase, Cochrane e CINAHL (via EBSCO) até junho de 2023. Foram incluídos os estudos clínicos relevantes nos quais terapias baseadas em células foram utilizadas para gerenciar a DA. O risco de viés foi avaliado utilizando os *checklists* do JBI, com base nos desenhos dos estudos.

**Resultados:**

Dos 1.014 registros examinados, foi incluído um total de cinco estudos com 70 indivíduos (incluindo 59 pacientes que receberam células-tronco e 11 controles de placebo). Em todos esses estudos, apesar da discrepância na origem das células-tronco, densidade celular e local de transplante, os objetivos de segurança foram alcançados. A injeção intracerebroventricular de ADSVF e UC-MSCs, a injeção intravenosa de Lomecel-B e a injeção bilateral dos hipocampos e *precuneus* direito de UC-MSCs não estão relacionadas a quaisquer preocupações significativas de segurança, de acordo com os cinco estudos incluídos. Os estudos também revelaram melhorias nos biomarcadores e resultados clínicos como um desfecho secundário. Três estudos não tinham grupos de controle e há preocupações quanto à semelhança dos grupos em outros. Além disso, há um risco considerável de viés em relação às escalas de avaliação de desfechos.

**Conclusão:**

As terapias baseadas em células são bem toleradas por pacientes com DA, o que enfatiza a necessidade de mais estudos randomizados cuidadosamente planejados para alcançar recomendações clínicas baseadas em evidências.

## INTRODUCTION

As the sixth leading cause of mortality^1^, and the third cause in older adults in the United States, Alzheimer’s Disease (AD) is the most common neurodegenerative disease^2^. Its prevalence in Europe is about 5.05%^3^. AD is characterized by progressive neurocognitive dysfunction due to the formation of extracellular amyloid plaques in the brain^4^. Currently, cholinesterase inhibitors and memantine — an antagonist of the N-Methyl-D-Aspartate — are used for boosting memory function in AD patients. some herbal components are also proposed to be effective; however, there is a lack of evidence for judgment in this regard^5-8^.

In addition to pharmacological interventions, stem cells (SCs), as a treatment approach, have enough potential to stop or even reverse the disease process and reduce the symptoms of AD^9^. The conventional belief that the adult central nervous system is incapable of neurogenesis has been disproved by the finding of neural SCs (NSCs)^10^. The theoretical ability of differentiation of SCs into neurons has been widely reported^11,12^. Also, evidence demonstrates the capability of transplants to become integrated into complex brain functions^13^. This potential makes AD one of the primary healthcare areas of cell therapy centers^14^.

SC replacement can cause the formation and maintenance of neural networks in the nervous system and prevent the progression of the disease by supporting the remaining cells and preventing the accumulation and production of toxic factors. Release of neurotrophins such as nerve growth factor (NGF), upregulating the expression of the anti-apoptotic factors, inhibition of activated microglia, as well as alleviating oxidative stress and inflammation are suggested as the possible mechanisms for the efficacy of mesenchymal SCs (MSCs)^15,16^. A systematic review of animal models of AD found excellent efficacy for MSCs in reducing cognitive deficits, which supports future clinical studies in this field. Based on nine preclinical studies incorporating 225 animals, MSCs-based treatment was associated with improved learning function and ameliorated the cognitive impairment, based on the Morris water maze test, in animal models of AD^17^.

Due to the lack of an up-to-date and exhaustive systematic review study on the clinical safety and efficacy of cell-based therapies in AD, such a study is necessary to reach a consensus on the scattered findings.

## METHODS

This study observed the Preferred Reporting Items for Systematic Reviews and Meta-Analyses (PRISMA) statement^18^ and Joanna Briggs Institute (JBI)’s methods for conducting systematic reviews^19^.

### Ethics approval

The research protocol was approved by the Ethics Committee of Tabriz University of Medical Sciences (ethics code: IR.TBZMED.VCR.REC.1398.338)

### Inclusion and exclusion criteria

Clinical studies in which cell-based therapies were used to manage patients with AD are included. All of the animal or in vitro studies, case reports, review articles, letters to editors, studies without efficacy or safety data, ongoing clinical trials, and withdrawn studies were excluded.

### Search strategy and study selection

A systematic search was conducted in June 2023 in PubMed, Medline (via Ovid), Embase; Cochrane, and Cumulative Index of Nursing and Allied Health Literature — CINAHL, via EBSCO) databases (by F-S.G.). Details about the search strategy are presented in Supplementary Material 1 In addition, the reference list of included studies, as well as the review studies, were manually checked for a comprehensive coverage of the published studies. After removing duplicate studies by EndNote 20 reference manager software, two authors screened the records in two title/abstract (S.S-S. and H.F.) and full text (R.M-H. and A.N.) stages, and studies that met the eligibility criteria were selected for inclusion. Disagreements were resolved by another researcher (H.S-P. or L.R.).

### Outcomes and data extraction

The desired outcomes were “efficacy of treatment” and “safety of treatment”. For this purpose, the necessary data, including the first author of the article, published year, study design, severity of AD, disease duration, number of participants, male-to-female ratio, age, type of transplanted SCs, cell density, SCs origin, transplantation zone, follow-up period, efficacy assessment scales, the efficacy of treatments, and safety data extracted by two authors (H.F., or H.K.) were collected using a data extraction table and double-checked by two other authors (M-S.H. and A.N.). The risk of bias (RoB) in the included studies was assessed using the JBI Critical appraisal tool^20^, by two authors (S.S-S and M-S.H.). Any disagreements during the mentioned stages were referred to another author (H.S-P. or L.R.).

## RESULTS

### Search results and screening

**Figure 1:**
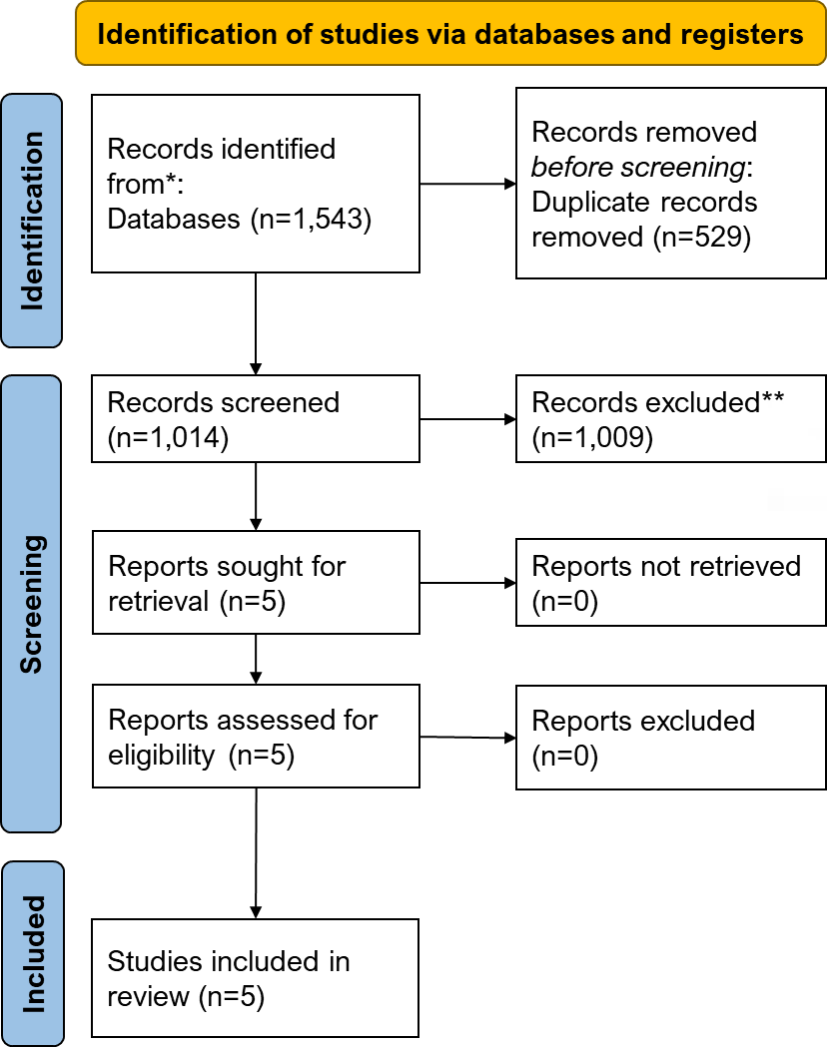
Preferred Reporting Items for Systematic Reviews and Meta-Analyses (PRISMA) flow diagram^18^.

Notes: *The total number across all databases; **No automation tools were used and all of the records were excluded by the researchers.

Overall, 1,543 articles were found through the database search. After duplicate removal, 1,014 studies were screened in the title/abstract stage, of which five were considered for further evaluation in the full-text stage, and all of these articles^21-25^ were included in the present systematic review (Figure 1).

### Characteristics of the studies

A total of five phase I studies with 70 individuals (including 59 patients receiving SCs and 11 placebo controls) were included in the present systematic review. The males constituted the majority of the included patients (37 males and 33 females). The mean age of participants in the included studies was demonstrated to be more than 60 years, with an approximate range of 61.6 to 75.5 years. Most studies (3 of 5) have utilized human umbilical cord-derived MSCs (UC-MSCs) to evaluate the tolerability of cell therapy among AD patients. Two other studies implemented adipose-derived stromal vascular fraction (ADSVF) and allogeneic MSC formulation (Lomecel-B). Eventually, the scales for outcome assessment were evaluated among the included studies, demonstrating somehow divergent scales, with the most prominent ones being Alzheimer’s Disease Assessment Scale-Cognitive Subscale (ADAS-Cog), Seoul-Instrumental Activities of Daily Living (S-IADL), Mini-Mental State Examination (MMSE), Consortium to Establish a Registry for AD (CERAD), Memory Performance Index (MPI), neuropsychiatric inventory (NPI), Clinician’s Interview-Based Impression of Change Plus Caregiver Input (CIBIC-Plus), as well as the cerebrospinal fluid (CSF) and plasma biomarkers, and imaging modalities including magnetic resonance imaging (MRI), positron emission tomography (PET) and computed tomography (CT). The characteristics of the studies are presented in Table 1.

**Table 1:** The characteristics and summary of the findings of studies on Alzheimer’s disease patients.

	P: patients				I: Intervention				O: outcome			
Study	Baseline MMSE	N	M:F	Mean age ± SD	Types of SCT	Cell density	Where cells derived	Transplantation zone	Intervention-outcome measurement interval	Outcome assessment scales	Summary of results	Cognitive outcomes
Kim et al.^24^	16.6±4.1	9	6:3	61.6±6.9	human UC-MSCs	(3, 6)×10^6^ cells	umbilical cord blood	bilateral hippocampi and right precuneus	4, 12 weeks, and 24 months	ADAS-Cog, S-IADL, MMSE, PiB-PET, FDG-PET, CSF biomarkers, brain CT and MRI	In the 12-week follow-up, wound pain (100%), headache (44.4%), dizziness (33.3%), delirium (33.3%), nausea (22.2%), and back pain (22.2%) were the most common adverse events, none of which were extended to 24 months. There were no structural abnormalities or immunological reactions.	Changes in ADAS-Cog, in the low-dose group, were 5.3±3.5 (week 12) and 20.0±9.9 (month 24); in the high-dose group, they were 3.5±5.6 (week 12) and 8.6±13.1 (month 24). Changes in S-IADL, in the low-dose group, were 1.7±4.0 (week 12) and 19.5±6.4 (month 24); in the high-dose group, they were 1.2±5.9 (week 12) and 12.0±6.0 (month 24). Changes in MMSE, in the low-dose group, were -1.7±0.6 (week 12) and -9.5±0.7 (month 24); in the high-dose group, they were 0.5±2.1 (week 12) and -8.4±5.6 (month 24).
Duma et al.^23^	Various	10	6:4	75.5±11.6	ADSVF	3.5–20 cc containing 4.05×10^5^ to 6.2×10^7^ cells/cc	liposuction	Intracerebroventricular (Ommaya reservoir implantation)	2 to 36 months	MPI, RBANS, NeuroQuant^®^ volumetric MRI, CSF biomarkers	There was no complication other than mild headache, or pain at the surgical sites, for less than 24 hours. Over eight months, 30% showed an improvement in CSF markers. Hippocampal volume increased in one of four assessed AD patients after 2 years of follow-up.	Over eight months, 80% of AD patients had stable or improved cognitive function, assessed by MPI.
Kim et al.^22^	21.1±3.82	9	3:6	62.7±7.93	human UC-MSCs	(1-3)×10^7^ cells/2 mL [three repeated injections]	umbilical cord blood	Intracerebroventricular (Ommaya reservoir implantation)	4,8, 12, 52, 104 weeks	ADAS-Cog, S-IADL, MMSE, NPI, CIBIC-Plus, CSF biomarkers, MRI, PiB-PET or florbetaben PET	Fever (100%), headache (77.8%), nausea (55.6%), vomiting (44.4%), myalgia or chills (22.2%), paresthesia on both arms and legs (22.2%) were the most commonly reported adverse events, all of which subsided within 36 hours. There were also three serious adverse events in two participants which required an extended hospitalization.	Changes in ADAS-Cog, in the low-dose group were 0.7±4.0, and in the high-dose group, were 2.3±5.0 (week 12) Changes in S-IADL, in the low-dose group were 2.0±2.0, and in the high-dose group were 1.5±3.0 (week 12) Changes in MMSE, in the low-dose group, were 0.0±2.0, and, in the high-dose group were 0.7±1.6 (week 12). Changes in NPI, in the low-dose group, were 1.3±2.3, and, in the high-dose group, were -1.7±5.8 (week 12).
Myeong et al. ^21^	21.6±1.39	6 (+3 placebo)	5:4	63.4±3.64	human UC-MSCs	3×10^7^ cells/2 mL	umbilical cord blood	Intracerebroventricular (Ommaya reservoir implantation)	24 hours	CSF pro-inflammatory cytokine	Injection was associated with increased CSF levels of TNF-α, IL-1β, IL-6, and CRP; however, bacterial culture results were negative.	-
Brody et al.^25^	20.45±1.46, 20.60±2.06, 20.70±2.26	25 (+ 8 placebo)	17:16	71.2±8.4	Lomecel-B	(2-10)×10^7^ cells	Bone marrow	single intravenous infusion	13, 26, 52 weeks	MMSE, ADAS-Cog-11, ADCS-ADL, ADRQL, GDS, NPI, QOL-AD, Trail Making Test, plasma biomarkers, MRI	There was only one treatment-emergent serious adverse event. The incidence of adverse events was lower in each Lomecel-B treatment arm. There were no amyloid-related imaging abnormalities.	Low-dose (but not high-dose) Lomecel-B arm was significantly better than the placebo at week 13 by 2.69±1.39 points in MMSE. Lomecel-B arms appeared more stable in ADAS-Cog-11, and the difference at week 26 between placebo and low-dose Lomecel-B arms, while not significant, was 5.68±3.66.

Abbreviations: N, number of patients; M:F, Male:Female; MMSE, Mini-Mental State Examination; AD, Alzheimer’s disease; ADAS-Cog, Alzheimer’s Disease Assessment Scale-Cognitive Subscale; S-IADL, Seoul-Instrumental Activities of Daily Living; CERAD, Consortium to Establish a Registry for Alzheimer’s Disease; MPI, Memory Performance Index; CSF, cerebrospinal fluid; UC-MSCs, umbilical cord blood-derived mesenchymal stem cells; ADSVF, adipose-derived stromal vascular fraction; RBANS, repeatable battery for the assessment of neuropsychological status; MSC, mesenchymal stem cells; NPI, neuropsychiatric inventory; ADCS-ADL, Alzheimer’s Disease Cooperative Study-Activities of Daily Living; ADRQL, Alzheimer’s Disease Cooperative Study-Activities of Daily Living; GDS, Geriatric Depression Scale; QOL-AD, Quality of Life in Alzheimer’s Disease; CIBIC-Plus, Clinician’s Interview-Based Impression of Change Plus Caregiver Input.

### Risk of bias assessments

Table 2 presents the details of RoB assessments based on the JBI critical appraisal tool. Three studies had no control groups and the other two studies did not report enough data to judge the similarity of the groups. Also, there is considerable RoB regarding the outcome assessment scales.

**Table 2:** Details of the risk of bias assessments based on the Joanna Briggs Institute Critical Appraisal tool for Quasi-experimental studies included in this systematic review.

Questions	Author (year)				
	Myeong et al.^21^	Brody et al.^25^	Kim et al.^22^	Duna et al.^23^	Kim et al.^24^
Q1: Is it clear in the study what the ‘cause’ is and what the ‘effect’ is (i.e. is there no confusion about which variable comes first)?	Y	Y	Y	Y	Y
Q2: Were the participants included in any similar comparisons?	U	U	N	U	N
Q3: Were the participants included in any comparisons receiving similar treatment/care, other than the exposure or intervention of interest?	U	U	N	N	N
Q4: Was there a control group?	Y	Y	N	N	N
Q5: Was the data analysis conducted with sufficient coverage of the identified sample?	Y	Y	Y	U	Y
Q6: Was follow-up complete? And, if not, were differences between groups in terms of their follow-up adequately described and analyzed?	N	Y	Y	N	Y
Q7: Were the outcomes of participants included in any comparisons measured in the same way?	Y	Y	Y	Y	Y
Q8: Were outcomes measured in a reliable way?	U	Y	U	U	U
Q9: Was appropriate statistical analysis used?	Y	U	N	U	N

Abbreviations: Y, Yes; N, No; U, Unclear.

### Results of individual studies

In a phase 1 clinical trial, nine individuals with mild-to-moderate AD (MMSE: 16.6±4.1) produced acceptable and secure outcomes. In this study, in addition to AChE-I, low (3.0*10^6^ cells/60 mL) and high (6.0*10^6^ cells/60 mL) doses of UC-MSCs were injected into the bilateral hippocampi and right precuneus and, during the 12-week follow-up period, there was no considerable safety issues and dose-limiting toxicity. There was no fever or cerebral hemorrhage in control CT scans. Surgical wound pain, headache, dizziness, delirium, nausea, and back pain were the adverse events and in the extended 24-month follow-up there was no adverse event. There were no tumor and subdural hemorrhages in 12-month and 24-month control MRIs. Regarding clinical outcomes, improved ADAS-Cog, S-IADL, and MMSE scores were evident^24^.

Four years later, Duma et al., in a 3-year phase 1 study, approved the safety of ADSVF injection (3.5–20 cc [median: 4 cc] containing 4.05×10^5^ to 6.2×10^7^ cells/cc) into the human brain ventricular system, receiving through an implanted reservoir or via ventriculoperitoneal shunts. The sample of this study includes ten AD patients with no other treatment options, and reported complications include acute hydrocephalus and severe meningismus after first injection. A decrease in tau protein and an increase in hippocampal volume were reported in two patients with eight injections, and an improved memory index was reported in 30% of the samples^23^.

Kim et al. performed another phase 1 clinical trial to assess the safety of three repeated intracerebroventricular injections of low (1.0*10^7^ cells/2 mL) and high (3.0*10^7^ cells/2 mL) doses of UC-MSCs on nine mild-to-moderate AD patients in 2021. Injections in this study were associated with three serious adverse events; however, there was no dose-limiting toxicity. Increased CSF levels of white blood cells (WBCs), fever, headache, nausea, and vomiting which all subsided within 36 hours were the most commonly reported adverse events in this study, and serious adverse events were limited to extended hospitalization by one day. There was no tumor development, hydrocephalus, or hemorrhage in the extended observation study for 36 months^22^. To delineate the cause of fever, researchers conducted another study and assessed the CSF level of multiple cytokines. Investigators demonstrated that transplantation of UC-MSCs was associated with increased levels of inflammation cytokines, including tumor necrosis factor-α (TNF-α), interleukin-1β (IL-1β), interleukin-6 (IL-6), and c-reactive protein (CRP) levels, with no bacterial source; therefore, it was concluded that the transient inflammatory response was due to the transplanted UC-MSCs^21^.

The most recent study was done by Brody et al. in 2023 and involved 33 mild AD patients. Eight patients received a placebo in this double-blind randomized controlled trials (RCT), and 25 underwent a single infusion of low (2.0*10^7^ cells) or high (1.0*10^8^ cells) doses of Lomecel-B, MSCs isolated from fresh bone marrow tissue. In this study, the primary safety endpoint was met, and significant improvement was achieved regarding the neurocognitive, imaging, and CSF biomarkers. In this study, treatment-emergent serious adverse events were observed in one patient in the high-dose group. The overall incidence of adverse events was lower in Lomecel-B groups in comparison to the placebo group and there were no amyloid-related imaging abnormalities^25^.

### Results of synthesis

The evidence regarding cell-based therapies in AD is mostly limited to safety assessments. Based on five included studies, bilateral hippocampi and right precuneus injection of UC-MSCs, intracerebroventricular injection of ADSVF and UC-MSCs, and intravenous injection of Lomecel-B are not associated with considerable safety issues. In addition, as a secondary outcome, studies suggested clinical and biomarker improvements, too.

## DISCUSSION

This systematic review was conducted to explore the evidence regarding the efficacy and safety of cell-based therapies in AD patients. Based on the limited available evidence, this procedure seems to be safe and well-tolerated by AD patients in different stages of the disease, and it can be associated with clinical improvements; however, these findings arose from phase 1 clinical trials with small sample size and there is a need for future well-designed RCTs for clinical recommendations in this regard.

The basis of neurodegenerative diseases’ pathogenesis is a progressive loss of function, structure, or number of neurons^26^. However, the complexity of associated underlying mechanisms prevents understanding the exact pathogenic processes in each disease. Also, the blood-brain barrier causes significant limitations in developing effective pharmacologic agents^27^. In this condition, regenerating neural tissue, providing neurotrophic support, alleviating neurodegeneration, and stabilizing the neuronal networks by SCs offer promising treatments for almost all neurodegenerative diseases^28^. SCs can help scientists in the treatment and better understanding of AD-related dementia mechanisms^29^.

SC therapy, commonly known as regenerative medicine, promotes the repair response of injured tissue^30^. It is routinely used for cancer and blood-related diseases^31^. SCs are unspecialized human body cells with the capacity for self-renewal, and can develop into different types of organism cells^32^. The pluripotency in SCs is a continuum that includes the spectrum from embryonic SCs to multi-, oligo- or unipotent cells^33^. MSCs, such as adipose-derived MSCs and bone marrow MSCs, are multipotent progenitor cells that can be isolated from multiple human tissues^34^ and used as a significant source of cells with regenerative and anti-inflammation potential^35,36^. SVF is also the initial product of adipose tissue^37^, excluding mature adipocytes^38^, which include heterogeneous cell populations, among them adipose-derived MSCs, endothelial cells, and macrophages^39^. NSCs are also the SCs of the nervous system, which can differentiate into neurons, astrocytes, and oligodendrocytes, three major cell types in the central nervous system^40^.

There is still a slight improvement in cell-based treatments for AD. Although the exact underlying mechanism of how SCs can boost the cognitive function of AD patients is still unclear^41^, recent studies have found neurogenesis and synaptogenesis as well as reducing Aβ accumulation potential of MCSs^42-44^. In addition, evidence supports the capability of different SCs to differentiate into cholinergic neurons^45^. Based on our findings, cell-based treatments were well-tolerated in AD patients, but confirming significant improvements in patients’ conditions needs more well-designed trials with larger sample sizes. Ongoing clinical trials may confirm or reject the current opinions. AstroStem is one of the ongoing trials in the phase 1/2 study. In this trial, the SCs extracted from the fatty tissue of patients and outcome measurement will be based on adverse events and cognitive function, behavior and mood, daily activity, and biomarkers^46^.

To the best of our knowledge, this study was the first systematic review to assess the efficacy and safety of cell-based therapies in AD. Comprehensive coverage of eligible studies, as well as PRISMA and JBI-guided methods, were the leading strengths of this study. On the other hand, excluding non-English papers was the limitation during the review process and the small number of included studies, small sample sizes, and lack of well-designed RCTs were the main limitations of the evidence.

In conclusion, cell-based therapies are well tolerated in patients with AD. Also, the treatments’ efficacy in reducing disease progression introduces cell-based therapy as a new therapeutic approach in AD; however, the limitations of the evidence highlight the need for future well-designed RCTs. Also, future studies should aim to find the best type and sources of cells, doses, and route of administration in each condition.
